# Multidisciplinary Training in Dermatology: Exploring the Spectrum of Board Certifications Among Physicians Practicing Dermatology in Texas

**DOI:** 10.7759/cureus.62839

**Published:** 2024-06-21

**Authors:** Rebecca H Lee, Jason Lee, Jonny Hatch, Lilian Zhan, Braden Van Alfen, Mark Conley, Tracy Zhao, Carlos Gomez-Meade

**Affiliations:** 1 Family Medicine, Kaiser Permanente, Anaheim, USA; 2 Family Medicine, Riverside University Health System Medical Center, Moreno Valley, USA; 3 Internal Medicine, Baylor Scott & White All Saints Medical Center, Fort Worth, USA; 4 Osteopathic Medicine, Sam Houston State University, Houston, USA; 5 Texas College of Osteopathic Medicine, University of North Texas Health Science Center, Fort Worth, USA; 6 Life Sciences, Utah Valley University, Orem, USA; 7 Mohs Surgery, Oklahoma Cancer Specialists Skin Cancer Center, Broken Arrow, USA

**Keywords:** specialty boards, multidisciplinary training, internal medicine, family medicine, dual board-certified, multiple residencies

## Abstract

Dermatology, a medical specialty focused on skin, hair, and nail conditions, often overlaps with various medical specialties. Although most physicians practicing dermatology are board-certified in dermatology, there are physicians practicing dermatology who are board-certified in a variety of different medical specialties. This cross-sectional study examines the board certifications of physicians who reported practicing dermatology in Texas. Data were sourced from the Texas Medical Board database updated in August 2023. The data showed that out of 1,614 practicing physicians declaring dermatology as a specialty in Texas, 1,080 (66.91%) physicians had one board certification, 200 (12.39%) had two, and 15 (0.93%) had three. Of the physicians with one board certification, 1,053 (97.5%) were board-certified in dermatology, with the remainder primarily being a mix of a variety of primary care specialties but also including other specialties. This study confirmed that while the vast majority of physicians practicing dermatology are board-certified in dermatology, there are opportunities for physicians board-certified in other specialties to practice dermatology as well.

## Introduction

A dermatologist specializes in conditions involving the skin, hair, and nails [1], which can encompass a large variety of diseases and intersect with other specialties. Dermatologists are often consulted in the inpatient and outpatient settings [2]. A survey of the Women’s Dermatologic Society found that 85% of responders reported working closely with different specialties, with the most common being obstetricians and gynecologists, rheumatologists, nutritionists, neurologists, internists, and cardiologists [3]. Skin conditions are especially prevalent in primary care. A retrospective study examining patient encounters with their primary care physician over two years showed that 36.5% of patients presented with at least one skin problem [4].

While the traditional route to becoming a dermatologist involves a postgraduate year of medicine (usually internal medicine, transitional year, or preliminary year), most internal medicine residencies offer only one week or less of dermatology training [5]. Family physicians may receive more dermatology-specific training depending on interest and program, but there are no specific requirements set by the ACGME [6]. Dermatology has historically been separated from medicine and vice versa, but the value in holistic and integrative care was recognized more than 20 years ago [7]. Now five combined internal medicine-dermatology residencies exist in the US for physicians to be able to sit for both board exams [8]. Fellowships such as pediatric dermatology have also been created to allow those interested to acquire additional training in dermatology [8].

Board certifications started in 1917 and are now often required for credentialing [9,10]. Physicians participate in required continuing education to stay up to date on the newest advances within their specialty [9,10]. Given the complex cases in dermatology and the overlap between different fields, we explored the percentage of physicians practicing dermatology who declared specialties in addition to dermatology or were board-certified in another specialty.

## Materials and methods

Design

This is a cross-sectional study. Data regarding declared board certifications among physicians who self-reported dermatology as a specialty they practice in were extracted from the Texas Medical Board (TMB) database in August 2023. The TMB is the state board governing medical professionals. It allows for patients to search for a provider and find their license status, educational background, or any prior disciplinary actions. There is also a section that is self-reported by the provider and not verified by the TMB such as specialty or practice address. Our data included this verified and unverified (self-reported) information.

Data collection

Inclusion criteria were physicians with an active medical license, including both medical doctors (MDs) and Doctors of Osteopathic Medicine (DOs), who reported that they were practicing in the field of dermatology (self-reported). This information was found by filtering for a “physician” under license type and “dermatology” under specialty. This yielded a list of physicians in alphabetical order. The primary and secondary specialty of each physician, as well as their first, second, and third board certification (if any), were manually compiled and recorded in a Google Sheet for ease of collaboration between authors. Once all names and specialty information were recorded, these data were then exported into a Microsoft Excel document for data manipulation.

Data manipulation

The individual physicians were first sorted into four different categories: those with zero, one, two, and three board certifications through the filter function on Excel. Within the categories of one, two, or three board certifications, physicians were further separated by their board certifications. Those with one board certification were separated by their specialty board and counted using the count function. Percentages of each board certification were calculated using the percentage function and rounded to the second decimal. Those with two or three board certifications were manually separated based on their unique combination of board certifications, such as those described above, each was counted using the count function, and the percentage was calculated using the percentage function of Excel and rounded to the second decimal. Board certifications were not organized by chronological order of achievement.

## Results

Table 1 summarizes the number of physicians with zero, one, two, or three board certifications. Out of 1,614 practicing physicians declaring dermatology as a specialty in Texas, 319 (19.76%) did not list a board certification, 1,080 (66.91%) declared one board certification, 200 (12.39%) declared two board certifications and 15 (0.93%) declared three board certifications (Table 1).

**Table 1:** Number of board certifications in physicians practicing dermatology

Number of Board Certifications	Number (1,614)	Percentage
None Listed	319	19.76%
1	1,080	66.91%
2	200	12.39%
3	15	0.93%

Table 2 depicts the specific board certifications listed in the 1,080 physicians who declared one board certification and the number of total physicians listing that board certification as their only board. Approximately 1,053 out of the 1,080 dermatologists (97%) with one board certification were boarded in dermatology (Table 2). Fifteen (1.39%) physicians listed family medicine as their board certification, which was the second most common (Table 2). Less common board certifications include pediatrics, internal medicine, emergency medicine, ophthalmology, pathology, and surgery.

**Table 2:** Board certifications of physicians practicing dermatology with one board certification

Board Certifications	Number (1,080)	Percentage
Dermatology	1,053	97.5%
Family Medicine	15	1.39%
Pediatrics	4	0.37%
Internal Medicine	3	0.28%
Emergency Medicine	2	0.19%
Ophthalmology	1	0.09%
Pathology	1	0.09%
Surgery	1	0.09%

Table 3 lists the specific board certifications in the 200 physicians who declared two board certifications and their respective percentages are listed based on a unique combination of board certifications. Similarly, Table 4 lists the specific board certifications in the 15 (0.93%) physicians who declared three board certifications and the percentages of each, again separated based on their unique combination of board certifications. Approximately 103 (51.50%) physicians declaring two board certifications have an additional fellowship board certification in dermatopathology (Table 3). The most common combination of triple board certifications was dermatology, pediatrics, and pediatric dermatopathology, which included six (40%) physicians (Table 4).

**Table 3:** Board certifications of physicians practicing dermatology with two board certifications OMT: osteopathic manipulative therapy

Board Certifications	Number (200)	Percentage
Dermatology + Dermatopathology	103	51.5%
Dermatology + Internal Medicine	38	19%
Dermatology + Pediatric Dermatology	24	12%
Dermatology + Pathology	12	6%
Dermatology + Pediatrics	7	3.5%
Dermatology + Clinical and Lab Dermatological Immunology	4	2%
Dermatology + Family Medicine	3	1.5%
Dermatology + Allergy and Immunology	2	1%
Dermatology + Emergency Medicine	1	0.5%
Dermatology + Ophthalmology	1	0.5%
Dermatology + Plastic Surgery	1	0.5%
Dermatology + Preventative Medicine	1	0.5%
Family Medicine + OMT	1	0.5%
Internal Medicine + Adolescent Medicine	1	0.5%
Pathology + Dermatopathology	1	0.5%

**Table 4:** Board certifications in physicians practicing dermatology with three board certifications

Board Certifications	Number (15)	Percentage
Dermatology + Pediatrics + Pediatric Dermatopathology	6	40%
Dermatology + Pathology + Dermatopathology	3	20%
Dermatology + Internal Medicine + Allergy and Immunology	1	6.67%
Dermatology + Internal Medicine + Dermatopathology	1	6.67%
Dermatology + Internal Medicine + Endocrinology	1	6.67%
Dermatology + Internal Medicine + Hematology	1	6.67%
Dermatology + Emergency Medicine + Pediatrics	1	6.67%
Pediatrics + Pediatric Dermatology + Adolescent Medicine	1	6.67%

## Discussion

Our study aimed to examine the scope of training of physicians who declared dermatology as their specialty in Texas by reviewing the quantity and variety of board certifications.

We found that the majority of physicians practicing dermatology in Texas had one board certification (n=1,080, 66.91%) and within those, the majority (n=1,053, 97.50%) were board-certified in dermatology. The remainder of those with one board certification consisted of primary care (e.g., family medicine, internal medicine, and pediatrics), emergency medicine, ophthalmology, surgery, and pathology. Approximately 12.39% had two board certifications. This included those that may have completed an additional fellowship after residency such as dermatopathology or pediatric dermatology. Dermatopathology was the most common additional board certification (n=103, 51.50%) within this group, and internal medicine was the second most common (n=38, 19%). Three board certifications were rare at 0.9% (n=3). 

Medical board certifications began in 1917 and by 2002, the American Board of Medical Specialties (comprising 24 specialties including dermatology) compiled a shared set of requirements for certification [9]. Board certifications help establish boundaries for individual specialties to practice within [9]. These boundaries may be one reason that many physicians are dual-certified. According to the American Board of Physician Specialties, the two primary reasons physicians choose to dual certify are to (1) advance their career and show expertise in multiple similar, yet unique specialties and (2) that some physicians learn enough about a new subspecialty throughout their career that they can obtain additional certifications and privileges [11].

In dermatology residency specifically, physicians receive comprehensive dermatologic training including dermatopathology and pediatric dermatology, and they are eligible to practice each [12]. However, some physicians choose to dedicate another year after residency to further specialize, potentially intending to teach, research, or practice specifically in that subspecialty. One study in 2017 found that dermatopathologists who were fellowship-trained were associated with a higher pathology exam volume [13]. These subspecialties can only be completed after one has obtained board certification in dermatology or pathology [12]. This would account for some of the dual board certifications.

Dual board certifications could also be driven by a change in interest or lifestyle. Approximately 27% of medical students apply for a different specialty than the original one they had preferred before starting medical school, most developing interests based on their experiences/rotations during medical school, which can vary greatly depending on the medical school [14].

A questionnaire completed by 3,571 resident physicians showed that 14.1% reported career choice regret [15]. Unlike other healthcare professions such as physician assistants/associates (PAs), physicians do not have the benefit of lateral mobility. A 2021 retrospective study found that in their first decade of work, one-third of PAs will change specialties at least once, with 28% leaving a primary care role for other specialties [16]. Completing another residency training could represent a demonstration of a similar trend.

Of the physicians who reported practicing dermatology in Texas with a single board certification, only 2.5% of those physicians held a board certification that was not dermatology. Most of the 2.5% were physicians with board certification in primary care (e.g., family medicine, internal medicine, and pediatrics). However, considering that 36.5% of patients seen in primary care were reported to have at least one skin concern, there may be a higher percentage of primary care physicians practicing dermatology but did not declare it as their primary specialty [4]. Dermatology access to the underserved population has posed a significant challenge and although teledermatology has been emerging as a potential solution, it is still limited considering the lack of a physical exam [17]. Utilizing primary care providers to help bridge this gap appears to be a possible remedy. A study by Castillo-Arenas examined the diagnostic agreement of primary care physicians and dermatologists and found that when it came to diagnosing a given skin condition, primary care physicians were able to do so with high specificity but low sensitivity. This suggests that primary care physicians were better qualified to rule out rather than provide an accurate diagnosis. An improvement in knowledge and skills training for primary care physicians regarding skin conditions would therefore improve the access and quality of dermatologic care [18].

The implementation of this idea has already proven to be successful, such as the pilot interventional study at the Veterans Affairs Palo Alto Health Care System. Primary care physicians underwent Internet Curriculum for Melanoma Early Detection (INFORMED) training, and their diagnostic accuracy for skin cancer or precancer was compared for 14 months before and after the intervention. An improvement in diagnostic accuracy was found in primary care physicians following additional training in skin cancer screening [19].

Limitations

The limitations of the study include that the data were limited to physicians registered on the Texas Medical Board as a dermatology specialist at the time of data extraction and therefore may not be a good representation of the overall dermatologist population in the United States. The chronological order of certifications was also not differentiated. It is therefore unclear if secondary board certifications were obtained before or after a dermatology residency (other than fellowship board certifications). Some dual board-certified dermatologists may have also completed a combination program that allowed them to sit for both boards and did not necessarily complete two separate residencies. We did not consider physicians who may have completed partial or additional residency training without taking the board exam. Physicians were also only able to report a maximum of three boards and, therefore, some may have more.

Additionally, board specialties and board certifications were self-reported by physicians and not verified by the Texas Medical Board. As such, inconsistencies are expected.

## Conclusions

Dermatology is a broad specialty that can intersect with various medical specialties. It is not uncommon for physicians practicing dermatology to pursue additional training beyond their dermatology residency whether through fellowships, combination programs, or separate residencies. However, the availability of specialized training options such as fellowships in dermatology for primary care of combination programs remains limited. This constraint may push those who desire further training to consider an additional residency, but multiple barriers exist to receiving additional training after completing an initial residency, the largest barrier being institutional funding. Nevertheless, dual-board-certified dermatologists can bring an important perspective to the field of dermatology. Moving forward, we believe it is crucial to address these barriers to promote greater accessibility to interdisciplinary training in dermatology, ensuring that this specialty continues to evolve and adapt.
